# Why apply for an intercalated research degree?

**DOI:** 10.1097/IJ9.0000000000000027

**Published:** 2017-06-08

**Authors:** Riaz Agha, Alex Fowler, Katharine Whitehurst, Shivanchan Rajmohan, Buket Gundogan, Kiron Koshy

**Affiliations:** a Guy's and St Thomas' NHS Foundation Trust; b Royal Devon and Exeter Hospital; c Imperial College London; d University College London; e Brighton and Sussex University Hospitals, UK

**Keywords:** Intercalated degree, Intercalated Bachelor of Science, BSc, iBSc, Intercalation, Research, Masters, MBPhD

## Abstract

Intercalated degrees are commonly undertaken as part of the medical undergraduate course. In this article, we discuss the advantages and disadvantages of intercalation, along with alternatives that could be considered.

## What is an intercalated degree?

The intercalated degree is an extra year’s study usually inserted between the second and third years of the medical course with the aim of studying a subject at greater depth and gaining a BSc, BA, or BMedSci degree.

The choice of degrees is vast, with some being heavily science oriented, such as Neuroscience, Anatomy, Physiology, Surgical Sciences. Others are clinically orientated, such as Primary Care and Child Health and others still are arts based, such as Global Health, History of Medicine, Psychology, and Business Management.

Approximately one-third of medical students in the UK medical schools undertake intercalated degrees. At some universities (Oxbridge, UCL, Imperial), this year is mandatory, but for others there is a big choice of whether *to intercalate or not to intercalate?*

## Things to consider

This is a year to study something that you’re interested in, gain a qualification, and learn new skills. Many students choose to do this at a different university to their medical degree.

There are many points to consider, summarized below as advantages and disadvantages:

### Advantages

#### Learning new skills

As a preclinical student, most study is rote learning to answer multiple choice questions. Most intercalated degrees, however, are examined through essays, and therefore new skills such as literature searching, critical appraisal, presentation skills, and scientific writing will be acquired. Students who have intercalated generally also have improved deep and strategic thinking^1^.

#### Research opportunity

Research is a vital part of improving patient care. Embarking on an intercalated degree can allow the opportunity to conduct research and progress the scientific knowledge base.

Research has shown that students who take intercalated degrees are likely to have more papers published in scientific journals, and to have gained research grants^2^,^3^.

#### Increases future options

Gaining a degree after 3 years of study opens up opportunities to pursue other professions (many of which don’t require a specific degree) and provides an exit strategy for those who no longer wish to pursue a career in medicine.

#### Pursuing speciality interests

The choice of which intercalated degree you do is yours. Here, you can learn more about what interests you, and show early commitment to the future specialty you’re considering. For a surgical career, degrees such as Anatomy and Surgical Science are good options. There is a lot of scope to focus on your interests, being able to choose your own modules, research project, and whom you work with. There is also the opportunity to work alongside nonmedical professionals, in an interdisciplinary team environment.

#### Career development

An intercalated degree looks good on a CV as it demonstrates extra skills, and that you have furthered your interests. It also helps with points for foundation applications and specialist training. The better the degree classification attained, the more points you’ll be provided. This is particularly important for competitive jobs and specialties.

#### Examination results

Research has indicated that medical undergraduates with an intercalated research degree performed better in medical undergraduate examinations^2^–^6^ and foundation applications^7^.

#### A year of being a normal student

An intercalated year generally involves fewer contact hours than medical years. This allows the time to pursue other interests, such as clubs and societies and activities outside of medicine.

### Disadvantages

The most common reasons students have opted not to intercalate are: the cost and the extra year of study^8^.

#### Cost

Some students are put off by another year of tuition fees. In the United Kingdom, since the rise of tuition fees to £9000, there has been a decrease in the uptake of intercalated degrees. Furthermore, you will be a year further from qualifying and earning a salary. On top of this, there are expenses through travel, living, and textbook costs.

There are ways of offsetting the cost of an intercalated year. There are many bursaries and grants available for students. Also, students that intercalate will receive an NHS bursary a year earlier: in the penultimate and final year, instead of just the final year. Many students can also fit in part-time work around their studies in this year, as contact hours are reduced.

#### Another year of studying

Some students have put off an intercalated year due to a lack of interest. It takes 5 years to complete the standard MBBS course and hence studying for an extra year requires students to pay careful consideration to motivational, career, and financial factors to make the right decision for them. The intercalated degree also comes hot on the heels of year 1 and 2 and thus students may be eager to progress into clinical years. Not all students have an interest in research, and therefore don’t want to pursue this extra year.

## Alternatives to an intercalated year

If you’ve decided not to intercalate, there are still other study options to further your interests:

- Defer the decision till after clinical medicine begins
  Some students have the option of continuing into clinical medicine and then doing an intercalated degree between the third and fourth or fourth and fifth years of study.
  PROS—more time to evaluate the different options, more time to gauge your own interests, your BSc can have a different focus, taking into account the clinical experiences acquired in the preceding year(s).
  CONS—this splits up clinical years and loses contact with patients at a time when your clinical skills are developing; difficulties in motivation.
- MBPhD
  Several medical schools offer the opportunity to do an integrated MBPhD program. This allows medical students to get a medical degree and a PhD in 7 to 9 years.
  PROS—If you are sure about a career in Academic Medicine then this is an option worth considering. This is because it takes less time than doing the degrees separately; the integrated nature of the degree minimizes the potential loss of clinical skills; funding and a stipend is usually provided for successful applicants.
  CONS—Few places available and often competitive.
- Masters Degree post qualification
  A part-time master’s degree done immediately after qualification over a 2-year period during your Foundation Years is another option.
  PROS—Doesn’t require a year out like an intercalated year; confers more “points” in future applications; likely to select a degree which is related more to your eventual specialty; likely to be more clinically relevant; networking potential and opportunities to launch parallel or portfolio careers.
  CONS—More expensive than intercalated degree; juggling study with full time foundation job.

## Links

- Database of intercalated course: http://www.intercalate.co.uk/.
- NHS medical careers: https://www.medicalcareers.nhs.uk/medical_students/intercalated_degrees.aspx.

## Summary

- The range of intercalated degree course available is vast.
- There are many advantages to intercalating, such as learning new skills and career development.
- Some students are worried about an extra year of debt; however, there are lots of opportunities for funding.
- There are many alternatives to taking an intercalated year.
